# Associations between prenatal blood metals and vitamins and cord blood peptide hormone concentrations

**DOI:** 10.1097/EE9.0000000000000275

**Published:** 2023-10-19

**Authors:** Anna R. Smith, Pi-I D. Lin, Sheryl L. Rifas-Shiman, Karen M. Switkowski, Abby F. Fleisch, Robert O. Wright, Brent Coull, Emily Oken, Marie-France Hivert, Andres Cardenas

**Affiliations:** a Department of Epidemiology and Population Health, Stanford Medicine, Stanford, California; b Division of Chronic Disease Research Across the Lifecourse, Department of Population Medicine, Harvard Medical School and Harvard Pilgrim Health Care Institute, Boston, Massachusetts; c Center for Outcomes Research and Evaluation, Maine Medical Center Research Institute, Portland, Maine; d Pediatric Endocrinology and Diabetes, Maine Medical Center, Portland, Maine; e Department of Environmental Medicine and Institute for Exposomic Research, Icahn School of Medicine at Mount Sinai, New York City, New York; f Department of Biostatistics, Harvard T.H. Chan School of Public Health, Harvard University, Boston, Massachusetts; g Diabetes Unit, Massachusetts General Hospital, Boston, Massachusetts

## Abstract

**Background:**

Nonessential metals have endocrine-disrupting properties, interfere with cellular processes, generate reactive oxygen, and deplete antioxidants, while essential metals and vitamins act as antioxidants. The extent to which prenatal metals and vitamins are associated with cord blood hormones involved in maternal and fetal metabolic and growth processes is unknown.

**Methods:**

We measured six nonessential (arsenic, barium, cadmium, cesium, lead, and mercury) and four essential (magnesium, manganese, selenium, and zinc) metals and trace elements, and two vitamins (B12 and folate) in first-trimester blood from participants in the longitudinal prebirth Project Viva cohort, who were recruited between 1999 and 2002 in eastern Massachusetts. We measured adiponectin, C-peptide, insulin-like growth factor (IGF)-1, IGF-2, IGF binding protein (IGFBP)-3, insulin, and leptin concentrations in cord blood (~n = 695). We used covariate-adjusted quantile g-computation for mixtures and linear regression for individual exposures to estimate associations with cord blood peptide hormones.

**Results:**

The essential metal mixture (magnesium, manganese, selenium, and zinc) was associated with higher IGF-1 (*β* = 3.20 ng/ml per quartile; 95% CI = 0.39, 6.01), IGF-2 (*β* = 10.93 ng/ml; 95% CI = 0.08, 21.79), and leptin (*β* = 1.03 ng/ml; 95% CI = 0.25, 1.80). Magnesium was associated with higher leptin (*β* = 2.90 ng/ml; 95% CI = 0.89, 4.91), while B12 was associated with lower adiponectin, IGF-2, and leptin but higher C-peptide. Other individual nonessential metals were associated with cord blood hormones.

**Conclusions:**

Our findings suggest that some prenatal metals and vitamins are associated with cord blood hormones, which may influence growth and development.

**What this study adds**

While there is evidence that prenatal metals and vitamins promote childhood growth and adiposity, there is limited information on the molecular mechanisms underlying these associations. This is the first epidemiological study to examine associations between prenatal metal and vitamin mixtures and cord blood peptide hormones, which are necessary for energy homeostasis and fetal growth and development. The study contributes to Environmental Epidemiology’s mission, as we used novel statistical methods to study mixtures of metals and vitamins relevant to public health in a large cohort study. We also utilized potential biomarkers of metabolic disease pathways and examined whether they were associated with select metals and vitamin concentrations during pregnancy. Future studies should confirm these findings and evaluate if the cord blood hormones mediate associations between prenatal metals and childhood adiposity.

## Introduction

Exposure to metals and trace elements in pregnancy is ubiquitous,^1^ and many cross the placental barrier and accumulate in fetal tissues. Some nonessential metals and trace elements have endocrine-disrupting properties and interfere with cellular processes by generating reactive oxygen species and depleting antioxidants,^2^ leading to inflammation, oxidative stress, and DNA damage.^3^ Conversely, essential metals at physiologic levels play an important role in physiological processes and could mitigate the effects of nonessential metals by acting as antioxidants.^4^ Other key prenatal nutrients, such as vitamin B12 and folate, are important methyl donors^5^ and are essential for the biosynthesis of macromolecules, DNA methylation, gene expression, in utero fetal development, and mitochondrial redox homeostasis.^6,7^ Similar to essential metals, vitamins may reduce the toxicity of nonessential metals. For example, in a randomized human trial of folate supplementation, arsenic detoxification was enhanced in the intervention arm of the trial.^8^ In another study, serum folate in pregnancy was inversely associated with blood mercury levels, independent of fish consumption.^9^

We previously observed that a prenatal mixture of nonessential metals was associated with higher childhood adiposity, whereas a prenatal mixture of essential metals was associated with lower childhood adiposity.^10^ One possible biological pathway that could mediate the observed associations between prenatal metals and childhood adiposity is through growth and metabolic-related peptide hormones. For example, higher cord blood adiponectin and leptin have been associated with early life weight gain and adiposity.^11–15^ A recent meta-analysis found that cord blood leptin was associated with greater adiposity at birth and lower adiposity up to 3 years of age, but not 5 years of age. In this study, there was a weak association between greater cord blood adiponectin and adiposity at birth.^16^ The insulin-like growth factor (IGF) axis, which plays a major role in the regulation of growth in utero and postnatally, includes three ligands (insulin, IGF-1, and IGF-2), at least four receptors, and IGF binding proteins (BP).^17^ These peptides have been implicated in child growth and development. Cord blood IGF-1 has been associated with higher neonatal birthweight, fat mass, and percent body fat.^18^ IGF-2 is the primary regulator of placental growth, nutrient transfer, and embryonic growth,^19,20^ and the IGF-2 gene is more abundantly expressed than IGF-1 in mid-gestation to late-gestation.^21^ Whereas IGF-1 appears to signal tissue growth due to nutrient sufficiency, IGF-2 may regulate late-gestation tissue-specific changes from cell differentiation and in response to adverse intrauterine conditions.^21^ Higher cord blood insulin and C-peptide have been associated with higher birthweight and insulin with higher fat mass at 1 year.^22^ Higher levels of leptin, IGF-1, IGF-2, and insulin were observed in cord blood samples of infants who were large for gestational age, compared with infants who were average for gestational age.^23^ The extent to which metals or nutrients influence these peptides is unknown. Thus, we aimed to conduct an untargeted analysis to evaluate associations between metals, nutrients, and peptide hormones as potential intermediary biomarkers for maternal and fetal metabolic and growth processes.

We leveraged a prospective cohort to examine the extent to which mixtures of- and individual- first-trimester blood metals and vitamins are associated with the cord blood peptide hormones leptin, adiponectin, C-peptide, insulin, IGF-1, IGF-2, and IGFBP-3. We hypothesized that essential and nonessential metals would be associated with altered concentrations of cord blood hormones, which may reflect early metabolic programming, and that the essential metals and nutrients would have associations in the opposite directions as the nonessential metals.

## Material and methods

### Study population and design

Between 1999 and 2002, we recruited pregnant persons in Project Viva, a prospective prebirth cohort study, during their first prenatal visit at Atrius Harvard Vanguard Medical Associates, a multispecialty group practice in eastern Massachusetts.^24^ Eligibility criteria included fluency in English, gestational age <22 weeks, singleton pregnancy, and plans to remain in the study area throughout gestation. Children were seen at visits from birth to adolescence.

Of 2128 participant-infant pairs, 28 participants contributed more than once due to multiple pregnancies. We used data from the first enrollment for this analysis (n = 2100) and measured first-trimester metals in maternal red blood cells (RBCs) among 1407 participants. Of these participants, 17 did not have mercury measurements. Of the 1390 participants with complete metal measurements, 695 had at least one cord blood hormone measured. We measured first-trimester vitamins, including B12 and folate, in 940 participants, and 484 had vitamins and at least one cord blood hormone measured (Figure S1, http://links.lww.com/EE/A245).

Participants provided written informed consent at recruitment and for postnatal follow-up visits. The Institutional Review Board of Harvard Pilgrim Health Care reviewed and approved all study protocols.

### Maternal first-trimester metal concentrations in red blood cells

We described metal measurements previously.^10,25^ Upon recruitment (median = 10 weeks gestation), a blood sample was obtained from participants and centrifuged. The RBC pellet was stored at −70°C before analysis, and we measured concentrations of metals in RBCs, except mercury, using triple quadrupole inductively coupled plasma mass spectrometry (ICP-MS) on a single run (Agilent 8800 ICP-MS-QQQ, Agilent Technologies, Inc., Wilmington, DE) in MS/MS mode with appropriate cell gases and internal standards. We measured mercury concentrations using a Direct Mercury Analyzer 80 (Milestone Inc., Sorisole, Italy).

We previously described quality control (QC) measures,^25^ which included calibration verification, procedural blanks, and repeat analysis of 2% of samples. We calculated intra-day coefficients of variation (CVs) using in-house QC pools at three concentrations before and after every 10 samples (N = 7). Intra-day CVs were <5% for analytes included in the analysis, except for selenium (<10%). Inter-day CV was <15%, except for concentrations near the limit of detection (LOD). The LOD and percent above the LOD for each metal were previously reported for the 1390 participants with complete metal measurements.^25^

We selected metals with an intra-class correlation (ICC) >0.70 among duplicates and >90% detection frequency across samples, including six nonessential metals and trace elements (arsenic, barium, cadmium, cesium, lead, and mercury), and four essential metals (magnesium, manganese, selenium, and zinc). Hereafter, we refer to all elements, including arsenic, as metals. Metal concentrations below the LOD were imputed using LOD/(√2). Metal concentrations were log2-transformed for statistical analyses to improve the interpretability of effect estimates.

### Maternal first-trimester vitamin concentrations in plasma

We measured B12 (pg/ml) and folate (ng/ml) concentrations in the plasma of the same first-trimester blood samples used to measure metals. We measured B12 using an electrochemiluminescence assay (Elecsys Vitamin B12 II, Roche Diagnostic, Indianapolis, IN), while we measured folate using an electrochemiluminescence binding assay (Elecsys Folate III, Roche Diagnostic). Both assays were conducted on the Roche Cobas 6000 system (Roche Diagnostics, Indianapolis, IN) and approved by the Food and Drug Administration for clinical use. The assay for B12 has day-to-day imprecision values of 7.6%, 4.4%, and 3.2% for concentrations of 203 pg/ml, 481 pg/ml, and 1499 pg/ml, respectively. The assay for folate has day-to-day imprecision values of 3.9%, 3.1%, and 2.0% for concentrations of 7.6 ng/ml, 14.3 ng/ml, and 19.2 ng/ml, respectively.

Hemolysis and lipemia were present in 13% and 5% of plasma blood samples, respectively. Two B12 samples were below the LOD (<150 pg), and we imputed them as the LOD/(√2). One B12 sample had a concentration above the assay upper limit of 4,000 pg/ml, and we imputed it as 4,000 pg/ml. We log2-transformed vitamin concentrations for statistical analyses.

### Cord blood hormones

The delivering clinician collected cord blood samples from the umbilical vein immediately after delivery. Whole blood was refrigerated for <24 hours, spun and aliquoted into samples, and stored in liquid nitrogen. We measured concentrations of IGF-1 (ng/ml), IGF-2 (ng/ml), and IGFBP-3 (ng/ml) from stored plasma by ELISA assay (IGF-1 and IGFBP-3: R&D Systems, Minneapolis, MN; IGF-2: Alpco Diagnostics, Salem, NH). We measured leptin (ng/ml) and adiponectin (μg/ml) by radioimmunoassay (Millipore Co, Darmstadt, Germany). We measured insulin (μU/ml) and C-peptide (ng/ml) by competitive electrochemiluminescence immunoassay (Roche Diagnostics, Indianapolis, IN). Lower limits of detection (intraassay CV; interassay CV) were previously reported.^26^

### Covariate assessment

Demographic characteristics were obtained through maternal self-report at study enrollment through a brief interview and questionnaire. We abstracted child sex from the delivery record. We used directed acyclic graphs to select model confounders based on prior knowledge and literature. These included maternal prepregnancy age, prepregnancy body mass index (BMI; calculated from self-reported weight and height), education (<college or college graduate), annual household income ($70,000 per year or less, >$70,000 per year), self-reported race and ethnicity as a proxy for shared differences in unmeasured variables, such as racism and marginalization (Asian, Black, Hispanic, White, or more than one race and ethnicity), smoking status (never, former, or during pregnancy), parity (nulliparous or >1), and infant assigned sex at birth (female or male). In models examining folate and B12, we adjusted for whether either hemolysis or lipemia was present in the plasma sample (n = 164) (yes/no) because these endogenous interferences could bias immunoassay results.^27^

### Statistical analyses: prenatal metal mixtures and cord blood hormones

We estimated associations of metal and vitamin mixtures with each cord blood hormone outcome using quantile g-computation.^28^ For the main models, we categorized metals in quartiles, treated the cord blood hormones (outcomes) as continuous values, and fit a linear model. We obtained the variance with a nonparametric bootstrap (B = 3000). We examined associations of the nonessential (arsenic, barium, cadmium, cesium, lead, and mercury) and essential (magnesium, manganese, selenium, and zinc) metal mixtures with each of the cord blood hormones, namely adiponectin, C-peptide, IGF-1, IGF-2, IGFBP-3, insulin, and leptin. We adjusted models for maternal prepregnancy age, BMI, education, household income, race, and ethnicity as a proxy for structural racism, smoking status, parity, infant sex, and any metal not included in the mixture of interest. As secondary analyses, we also tested associations between the essential metal and vitamin mixture (magnesium, manganese, selenium, zinc, B12, and folate) and each of the cord blood hormones, adjusting for the same covariates as main models and whether there was hemolysis or lipemia in the plasma sample. In models with IGF-1 or IGF-2 as the outcome, we ran additional models adjusting for IGFBP-3 to estimate associations independent of binding protein. We report estimates and 95% bootstrap confidence intervals (95% CI) for the difference in cord blood hormone for one quartile increase in metal mixture, conditional on covariates.

### Statistical analyses: individual prenatal metals and vitamins and cord blood hormones

We used adjusted multivariable linear regression models to test associations between individual metal concentrations and the cord blood hormones, adjusting for the same covariates as the mixture models. We tested associations of folate and B12 concentrations with each of the cord blood hormones, adjusting for covariates, as well as whether hemolysis or lipemia was observed. In models with IGF-1 or IGF-2 as the outcome, we conducted additional analyses adjusting for IGFBP-3 concentration as an indicator of free (unbound) IGF-1 or 2. We report estimates and 95% CIs for differences in each cord blood hormone associated with a two-fold increase in metal or vitamin concentration.

All analyses were conducted in R (version 4.1.0; R Development Core Team, Vienna, Austria).

## Results

### Population characteristics

Mean maternal age was 32.2 years (SD = 4.7 years) and 52.8% of offspring were female. Most participants had normal prepregnancy BMI (58.4%), never smoked (68.4%), and had at least a college education (66.3%) and high income (>USD$ 70,000 per year) (58.9%) (Table 1). The demographic variables of participants in this study subset were similar to those of the full cohort.^24^

**Table 1.** Characteristics of mother-child pairs from the Project Viva cohort with prenatal metal measurements who were included in at least one of the umbilical cord blood hormone analyses (n = 695)

Demographic characteristics		*N* (%) or mean (standard deviation)
Overall^a^		695
Maternal age (years)		32.2 (4.7)
Maternal prepregnancy body mass index (kg/m²)		
	Underweight (<18.5)	24 (3.5)
	Normal (18.5–<25.0)	406 (58.4)
	Overweight (25.0–<30.0)	153 (22.0)
	Obesity (≥30.0)	112 (16.1)
Maternal race and ethnicity		
	Asian	26 (3.7)
	Black	85 (12.2)
	Hispanic	45 (6.5)
	More than one race and ethnicity	27 (3.9)
	White	512 (73.7)
Maternal education		
	Less than college	234 (33.7)
	College graduate	461 (66.3)
Household income		
	≤$70,000 per year	286 (41.2)
	>$70,000 per year	409 (58.9)
Maternal smoking		
	Never	475 (68.4)
	Former	134 (19.3)
	During pregnancy	86 (12.4)
Parity		
	Nulliparous	328 (47.2)
	One or more	367 (52.8)
Assigned child sex at birth		
	Male	367 (52.8)
	Female	328 (47.2)

a Data were complete for all participants.

### Metal and vitamin distributions

Table S1, http://links.lww.com/EE/A245 describes the distributions of nonessential and essential metals in first-trimester RBCs, and Table S2, http://links.lww.com/EE/A245 shows the distribution of vitamins in the first-trimester plasma. Of the 484 participants with measured vitamin concentrations, no participants have a folate concentration lower than the clinical reference range, while 5 (1.0%) participants have a vitamin B12 concentration lower than the clinical reference range. The strongest correlations observed were between arsenic and mercury (*r* = 0.52) and magnesium and selenium (*r* = 0.44). Folate and B12 were moderately correlated (*r* = 0.22) (Figure S2, http://links.lww.com/EE/A245). Median RBC concentrations of all essential metals were within reference ranges, and median RBC concentrations of cadmium, lead, and mercury were similar to those in whole blood from subsets of female National Health and Nutrition Examination Survey participants, as illustrated in our prior studies.^10,25^

### Cord blood hormone distributions

Cord blood hormone distributions are described in Table 2. Hormones were positively and moderately correlated with one another (*r* < 0.5), except for insulin and C-peptide (*r* = 0.81), IGFBP-3 and IGF-1 (*r* = 0.67) or IGF-2 (*r* = 0.66) (Figure S3, http://links.lww.com/EE/A245). Table S3, http://links.lww.com/EE/A245 summarizes the origin and function of the hormones during pregnancy.

**Table 2.** Umbilical cord blood hormone summary measures among children from the Project Viva cohort with prenatal first-trimester metal concentrations

Cord blood hormone	Sample size	Median (IQR)	Minimum	Maximum
Adiponectin (μg/ml)	613	29.59 (8.69)	2.95	42.59
C-peptide (ng/ml)	673	0.92 (0.64)	0.03	7.63
Insulin (μU/ml)	673	4.67 (5.27)	0.22	93.87
IGF-1 (ng/ml)	674	52.38 (33.25)	7.44	171.27
IGF-2 (ng/ml)	674	399.60 (115.80)	102.80	740.90
IGFBP-3 (ng/ml)	674	1042.08 (392.65)	290.30	2819.90
Leptin (ng/ml)	590	6.97 (8.40)	0.48	39.96

IGF-1 indicates insulin-like growth factor 1; IGF-2, insulin-like growth factor 2; IGFBP-3, insulin-like growth factor binding protein-3; IQR, interquartile range.

### Associations between prenatal metal mixtures and cord blood hormones

In adjusted quantile g-computation analyses, a quartile increase in the essential metal mixture was associated with higher IGF-1 (*β* = 3.20 ng/ml per quartile; 95% CI = 0.39, 6.01), IGF-2 (*β* = 10.93 ng/ml; 95% CI = 0.08, 21.79), and leptin (*β* = 1.03 ng/ml; 95% CI = 0.25, 1.80) (Table 3). Manganese had the highest weight for the proportion of the positive association with IGF-1 and IGF-2, while selenium and magnesium had the highest weights for the proportion of the positive association with leptin (Figure S4, http://links.lww.com/EE/A245). The essential metal mixture was not significantly associated with adiponectin, C-peptide, insulin, or IGFBP-3, and the nonessential metal mixture was not significantly associated with any of the cord blood hormones (Table 3). When adjusted for IGFBP-3, the associations between the essential metal mixture and IGF-1 (*β* = 1.29 ng/ml; 95% CI = −0.72, 3.30) and IGF-2 (*β* = 3.13 ng/ml; 95% CI = −4.27, 10.52) were attenuated (Table 3).

**Table 3.** Quantile g-computation adjusted estimates for difference in each outcome for a one quartile increase in the nonessential or essential metal mixture

Outcome	*n*	Nonessential metal mixture^a^ [β (95% bootstrap CI)]	Essential metal mixture^b^ [β (95% bootstrap CI)]
Adiponectin (μg/ml)	613	0.25 (−0.90, 1.40)	−0.31 (−1.18, 0.56)
C-peptide (ng/ml)	673	−0.10 (−0.22, 0.01)	0.02 (−0.05, 0.09)
Insulin (μU/ml)	673	−0.47 (−1.78, 0.84)	−0.18 (−0.91, 0.56)
IGF-1 (ng/ml)	674	−0.62 (−4.04, 2.79)	3.20 (0.39, 6.01)
IGF-1 (ng/ml)^c^	674	−0.71 (−3.29, 1.87)	1.29 (−0.72, 3.30)
IGF-2 (ng/ml)	674	−10.05 (−25.36, 5.25)	10.93 (0.08, 21.79)
IGF-2 (ng/ml)^c^	674	−10.40 (−22.25, 1.44)	3.13 (−4.27, 10.52)
IGFBP-3 (ng/ml)	674	1.67 (−45.90, 49.24)	37.44 (−0.78, 75.65)
Leptin (ng/ml)	590	−0.53 (−1.59, 0.53)	1.03 (0.25, 1.80)

CI indicates confidence interval; IGF-1, insulin-like growth factor 1; IGF-2, insulin-like growth factor 2; IGFBP-3, insulin-like growth factor binding protein-3; N, sample size.

a Quantile g-computation model assessed difference in each outcome for one quartile increase in nonessential metals (arsenic, barium, cadmium, cesium, lead, and mercury), conditional on the covariates maternal age, prepregnancy body mass index, race and ethnicity, education, household income, smoking status, parity, child sex, and the essential metals.

b Quantile g-computation model assessed difference in each outcome for one quartile increase in essential metals (magnesium, manganese, selenium, and zinc), conditional on the covariates maternal age, prepregnancy body mass index, race and ethnicity, education, household income, smoking status, parity, child sex, and the nonessential metals.

c Quantile g-computation model assessed difference in each outcome for one quartile increase in nonessential or essential metals, conditional on the covariates maternal age, prepregnancy body mass index, race and ethnicity, education, household income, smoking status, parity, child sex, the nonessential or essential metals, and IGFBP-3.

Higher levels of the essential metal and vitamin (folate and B12) mixture were associated with higher IGF-1 (*β* = 5.35 ng/ml; 95% CI = 1.52, 9.19) and IGFBP-3 (*β* = 54.36 ng/ml; 95% CI = 0.38, 108.33) (Table S4, http://links.lww.com/EE/A245). When we adjusted for IGFBP-3, the association between the mixture and IGF-1 was attenuated (*β* = 2.66 ng/ml; 95% CI = −0.27, 5.58) (Table S4, http://links.lww.com/EE/A245).

### Associations between individual prenatal metals and vitamins and cord blood hormones

In linear regression analysis, a doubling in barium was associated with higher IGF-1 (*β* = 1.63 ng/ml; 95% CI = 0.24, 3.01) (Table 4). When we adjusted for IGFBP-3, the association between barium and IGF-1 was attenuated (*β* = 0.78 ng/ml; 95% CI = −0.24, 1.80) (Table S5, http://links.lww.com/EE/A245). Cadmium was associated with lower C-peptide (*β* = −0.04 ng/ml; 95% CI = −0.09, −0.00) and IGF-2 (*β* = −8.41; 95% CI = −15.26, −1.55). When we adjusted for IGFBP-3, the association between cadmium and IGF-2 was also attenuated (*β* = −4.20 ng/ml; 95% CI = −9.17, 0.77) (Table S5, http://links.lww.com/EE/A245). Cesium was associated with higher C-peptide (*β* = 0.11 ng/ml; 95% CI = 0.02, 0.21), while lead was associated with lower C-peptide levels (*β* = −0.11 ng/ml; 95% CI = −0.18, −0.03) and magnesium was associated with higher leptin (*β* = 2.90 ng/ml; 95% CI = 0.89, 4.91). We observed no associations between arsenic, mercury, manganese, selenium, or zinc and the cord blood hormones (Table 4).

**Table 4.** Multivariable linear regression results for the association between first-trimester red blood cell concentrations of each metal and plasma concentrations of each vitamin individually and umbilical cord blood hormones

Metal^a^	Adiponectin (μg/ml) [β (95% CI)]^b^	C-peptide (ng/ml) [β (95% CI)]^b^	Insulin (μU/ml) [β (95% CI)]^b^	IGF-1 (ng/ml) [β (95% CI)]^b^	IGF-2 (ng/ml) [β (95% CI)]^b^	IGFBP-3 (ng/ml) [β (95% CI)]^b^	Leptin (ng/ml) [β (95% CI)]^b^
	n = 613	n = 673	n = 673	n = 674	n = 674	n = 674	n = 590
As	−0.11 (−0.50, 0.27)	−0.01 (−0.04, 0.02)	−0.05 (−0.39, 0.29)	−0.68 (−1.92, 0.56)	−1.53 (−6.50, 3.44)	−1.49 (−17.94, 14.97)	−0.01 (−0.38, 0.36)
Ba	0.19 (−0.25, 0.62)	0.03 (−0.00, 0.07)	0.19 (−0.20, 0.57)	1.63 (0.24, 3.01)	3.61 (−1.94, 9.17)	16.60 (−1.76, 34.96)	0.09 (−0.32, 0.50)
Cd	−0.04 (−0.57, 0.49)	−0.04 (−0.09, −0.00)	−0.10 (−0.57, 0.37)	−0.94 (−2.67, 0.78)	−8.41 (−15.26, −1.55)	−20.19 (−42.94, 2.56)	−0.07 (−0.58, 0.44)
Cs	0.09 (−1.10, 1.28)	0.11 (0.02, 0.21)	0.63 (−0.40, 1.65)	0.77 (−2.97, 4.52)	−1.50 (−16.46, 13.45)	23.56 (−25.93, 73.05)	−0.04 (−1.17, 1.10)
Hg	0.06 (−0.32, 0.44)	0.00 (−0.04, 0.03)	−0.07 (−0.40, 0.26)	0.05 (−1.15, 1.25)	−0.96 (−5.77, 3.85)	6.08 (−9.83, 21.99)	−0.24 (−0.60, 0.13)
Pb	−0.05 (−1.00, 0.89)	−0.11 (−0.18, −0.03)	−0.55 (−1.38, 0.28)	−0.22 (−3.24, 2.80)	−3.14 (−15.21, 8.92)	0.53 (−39.43, 40.49)	−0.66 (−1.56, 0.23)
Mg	−0.17 (−2.31, 1.97)	0.01 (−0.16, 0.18)	−0.14 (−2.01, 1.73)	4.45 (−2.34, 11.25)	11.93 (−15.26, 39.11)	73.49 (−16.34, 163.35)	2.90 (0.89, 4.91)
Mn	0.20 (−0.63, 1.04)	0.03 (−0.03, 0.09)	0.38 (−0.29, 1.05)	1.48 (−0.96, 3.93)	8.83 (−0.93, 18.60)	11.82 (−20.56, 44.20)	0.43 (−0.36, 1.23)
Se	−0.05 (−2.05, 1.94)	0.08 (−0.08, 0.24)	0.04 (−1.72, 1.80)	2.56 (−3.84, 8.96)	0.20 (−25.40, 25.81)	26.95 (−57.79, 111.69)	1.89 (−0.06, 3.84)
Zn	−1.15 (−3.39, 1.09)	−0.15 (−0.32, 0.03)	−1.63 (−3.53, 0.27)	0.43 (−6.52, 7.37)	−8.46 (−36.22, 19.29)	8.09 (−83.83, 100.00)	0.61 (−1.54, 2.77)
Vitamin	[β (95% CI)]^c^	[β (95% CI)]^c^	[β (95% CI)]^c^	[β (95% CI)]^c^	[β (95% CI)]^c^	[β (95% CI)]^c^	[β (95% CI)]^c^
	n = 448	n = 467	n = 467	n = 467	n = 467	n = 467	n = 443
B12	−−1.95 (−3.23, −0.68)	0.11 (0.00, 0.21)	0.74 (−0.43, 1.91)	−0.06 (−4.08, 3.97)	−16.81 (−32.76, −0.87)	−41.12 (−94.69, 12.44)	−1.40 (−2.59, −0.22)
Folate	0.16 (−0.60, 0.93)	0.06 (−0.01, 0.13)	0.41 (−0.33, 1.16)	1.32 (−1.24, 3.87)	4.61 (−5.58, 14.79)	14.25 (−19.90, 48.40)	0.64 (−0.07, 1.36)

As indicates arsenic; B12, vitamin B12; Ba, barium; Cd, cadmium; Cs, cesium; Hg, mercury; Pb, lead; Mg, magnesium; Mn, manganese; Se, selenium; Zn, zinc.

a Metals and nutrients were log_2_-transformed in the analyses.

b Linear regression models were adjusted for maternal age, prepregnancy body mass index, race and ethnicity, education, income, smoking during pregnancy, parity, and child sex.

c Linear regression models were adjusted for maternal age, prepregnancy body mass index, race and ethnicity, education, income, smoking during pregnancy, parity, child sex, and whether hemolysis and/or lipemia was observed in plasma sample.

In individual models, a doubling in B12 was associated with lower adiponectin (*β* = −1.95 μg/ml; 95% CI = −3.23, −0.68), IGF-2 (*β* = −16.81 ng/ml; 95% CI = −32.76, −0.87), and leptin (*β* = −1.40 ng/ml; 95% CI = −2.59, −0.22), as well as higher C-peptide (*β* = 0.11 ng/ml; 95% CI = 0.00, 0.21). When we adjusted for IGFBP-3, the association between vitamin B12 and IGF-2 was attenuated (*β* = −8.20 ng/ml; 95% CI = −19.57, 3.17) (Table S6, http://links.lww.com/EE/A245). We observed no associations between folate concentration and any of the cord blood hormones.

## Discussion

In this study, the mixture of prenatal blood essential metals was associated with higher IGF-1, IGF-2, and leptin concentrations in cord blood. Manganese had the highest weight for the proportion of the positive association with IGF-1 and IGF-2, while selenium and magnesium had the highest weights for the proportion of the positive association with leptin. Consistently, in individual models, magnesium was associated with higher leptin. Although no other essential metals were individually associated with the cord blood peptide hormones, directions of association were similar to those in the mixture models. Vitamin B12 was associated with higher C-peptides. We also observed associations of B12 with lower cord blood leptin, adiponectin, and IGF-2. We did not observe associations between the mixture of the nonessential metals and cord blood peptide hormones, but in analyses of individual metals, cadmium and lead were associated with lower C-peptide, and cadmium was associated with lower IGF-2. We also observed associations between barium with higher IGF-1 and cesium with higher C-peptide. The associations between metals and vitamins and the IGFs were attenuated after adjusting for IGFBP-3, indicating that the exposures are associated with total but not free IGFs.

In our study, the essential metal mixture of magnesium, manganese, selenium, and zinc was associated with higher levels of IGF-1, IGF-2, and leptin. In individual metal models, higher prenatal magnesium was associated with higher leptin. Although there are no epidemiological studies to our knowledge that have assessed associations between these essential metals and cord blood peptide hormones, an animal study in Wistar/NIN rats found that prenatal magnesium restriction resulted in lower plasma leptin and higher percent body fat in the offspring, compared with rats without prenatal magnesium restriction.^29^ The study had major differences in species, tissue, timing of peptide hormone analysis, and exposure magnitude. However, it provides support for the role of prenatal magnesium in potentially altering hormones related to metabolic function early in life.

We also observed associations of higher prenatal vitamin B12 with higher cord blood C-peptide and lower cord blood leptin, adiponectin, and IGF-2. The greatest magnitude of association was between vitamin B12 and lower IGF-2. Both vitamin B12 and folate play a key role in one-carbon metabolism. In studies using a C57BL/6 mouse model, an altered ratio of dietary vitamin B12 and folic acid during pregnancy influenced the expression of imprinting loci, including H19/IGF-2, IGF2R, and KCNQOT1, in fetal tissues,^30–32^ which suggests our finding could be driven by epigenetic programming. We did not examine associations between the ratio of vitamin B12 and folic acid in our study, as no participants had folic acid below the clinical reference range. The associations between vitamin B12 and the cord blood hormones appeared to be driven by participants with high levels of vitamin B12, although we could not assess this further because there is no clinically defined level of vitamin B12 excess in pregnancy. In another Project Viva study, higher second-trimester protein intake was associated with lower IGF-2.^19^ We did not adjust for this variable in our study because it is a post-exposure variable. However, it is possible our findings are reflective of differences in diet since plasma B12 is most commonly derived from prenatal vitamin or protein intake.

Contrary to our hypothesis, prenatal folate was not associated with any of the cord blood hormones. One study found that folic acid supplementation during the embryonic period is associated with *IGF-2* DNA methylation in children,^33^ while another study found no differences between folic acid supplementation and *IGF-2* DNA methylation levels in infants.^34^ Although we would thus expect to observe a relationship between prenatal folate and cord blood IGF-2, it is possible we did not find associations because maternal folate levels in our cohort were high, likely due to high prenatal vitamin intake.^35^

In our study, cadmium and lead were associated with lower C-peptide, and cadmium was associated with lower cord blood IGF-2. Epidemiological studies have found associations between prenatal blood cadmium and lower crown-heal length and placental thickness,^36^lower birthweight,^37–39^ and increased odds of infants being born small for gestational age.^38^ In a United States longitudinal birth cohort study, prenatal cadmium has been associated with lower placenta IGF-2 and IGF-2-AS mRNA expression.^40^ The *IGF-2* gene is located within a cluster of imprinted genes and encodes a fetal and placental growth factor affecting birth weight.^41^ It has been hypothesized that cadmium may exhibit toxicity on growth and development by altering the expression of placental imprinted genes,^40^ although this was not evaluated in our study. In the Project Viva cohort, prenatal first trimester lead was associated with lower birthweight.^39^ Although there are no prior studies evaluating prenatal lead and C-peptide, our finding is biologically relevant, as C-peptide reflects the insulin-secretory activity of pancreatic *β*-cells, which modulates fetal growth,^42^ and lead is thought to interfere with the insulin signaling pathway due to its induction of oxidative stress.^43^

Our finding of no association between the nonessential metals and leptin is inconsistent with the existing literature. There is mouse model evidence that leptin and adiponectin expression are reduced in response to cadmium.^44^ In addition, a prospective cohort study in Canada found an association between prenatal blood cadmium (averaged between first and third trimester), and higher cord blood leptin in male but not female infants. In addition, prenatal lead was associated with increased odds of high cord blood leptin (≥90% vs. ≤10%).^45^ It is possible that our findings differed due to variations in the timing of metal measurement (first trimester vs. first and third-trimester average) or the larger sample size in the cohort from Canada.

Few epidemiological studies have examined associations between prenatal metals and hormones in early childhood, although it is difficult to extrapolate our findings in cord blood to those in early childhood, as cord blood hormonal levels may have different biological significance. In a prospective birth cohort in Mexico City, Mexico, second-trimester whole blood arsenic was associated with lower leptin in children (4–6 years).^46^ One prior longitudinal study examined associations between urinary arsenic and plasma IGF-1 in children from rural Bangladesh and found that concurrent arsenic exposure was associated with lower IGF-1 at a mean age of 4.5 years, while maternal urinary arsenic was associated with lower IGF-1 at birth, but not at 4.5 years.^47^ Another longitudinal study examining 9-year-old children from rural Bangladesh found that prenatal arsenic, but not current arsenic levels, was associated with higher IGFBP-3, which transports over 75% of circulating IGF-1,^48^ but no associations were observed with IGF-1.^49^ Our study is limited by the measurements of arsenic in blood which reflects both inorganic and potentially less toxic organic species.

There are few studies that have examined prenatal mixtures of metals and vitamins and their associations with cord blood hormones. While other studies have used whole blood, urinary, or toenail metal measurements as alternatives, red blood cell metal measurements are good indicators of overall metal burden for most metals, and their utility has been previously discussed.^50^ One study limitation includes the potential that some of the associations were observed by chance. Our findings may not generalize to all pregnant persons in the United States, as our population is limited geographically in Eastern Massachusetts, has access to health care, and is predominantly White with high education and income levels. In addition, our study population, on average, had high plasma levels of folate and vitamin B12, which is likely secondary to the high uptake of prenatal supplements in the Project Viva cohort.^35^

Finally, we did not assess the longitudinal trajectory of the hormones during pregnancy. In a prior study, higher IGF-I and IGFBP-3 concentrations in early gestation were associated with a higher risk of developing gestational diabetes during pregnancy. However, at the time of birth, the levels of both peptides did not distinguish between pregnancies with and without gestational diabetes.^51^ Similarly, we did not adjust for gestational diabetes or hypertensive disorders of pregnancy, which can lead to infants having a weight large for gestational age because these maternal outcomes could be on the causal pathway, and our analysis included participants with and without gestational diabetes and/or hypertensive disorders of pregnancy.

Given that few studies have examined prenatal metal and nutrient mixtures in relation to cord blood hormones, future studies are warranted, for example, to examine associations between metals and vitamins during each of the trimesters of pregnancy and cord blood hormones. It would also be useful to study these associations in populations with different exposure distributions of folate and vitamin B12. Such studies will help confirm the biological mechanisms that link prenatal metal and nutrient exposures to postnatal growth, adiposity, and cardiometabolic health, which have been studied in experimental studies but less in epidemiological studies. Similarly, future studies examining whether growth and metabolism-related hormone concentrations in cord blood mediate associations between prenatal metal exposures and childhood adiposity outcomes would be useful. The results could inform the development of future biomarkers of postnatal disease processes at birth for early intervention.

## ACKNOWLEDGMENTS

We thank the participants and staff of Project Viva.

## Supplementary Material
