# Supplementary material for: Associations between prenatal blood metals and vitamins and cord blood peptide hormone concentrations
Source: Environ Epidemiol. 2023 Oct 19;7(6):e275. doi: 10.1097/EE9.0000000000000275 (PMC11031200; doi:10.1097/EE9.0000000000000275)
Supplement: Supplementary file 1 [file ee9-7-e275-s001.docx]

**Supporting Information**

**Table S1.** Distribution of metals in first trimester red blood cells for participants with all metals measurements and included in at least one of the cord blood peptide hormone analyses (*N*=695).

| Metal  (ng/g) | Mean | 25^th^ percentile | 50^th^ percentile | 75^th^ percentile | Minimum | Maximum | | % >LOD | | LOD (ng/g) |
| --- | --- | --- | --- | --- | --- | --- | --- | --- | --- | --- |
| Arsenic | 1.20 | 0.38 | 0.83 | 1.50 | 0.10 | | 12.40 | | 94 | 0.15 |
| Barium | 5.57 | 2.06 | 3.16 | 5.93 | 0.29 | | 59.20 | | 99 | 0.41 |
| Cadmium | 0.50 | 0.26 | 0.38 | 0.54 | 0.04 | | 6.94 | | 100 | 0.06 |
| Cesium | 2.66 | 2.02 | 2.51 | 3.14 | 0.84 | | 6.63 | | 100 | 0.06 |
| Lead | 19.61 | 13.40 | 17.50 | 23.65 | 4.11 | | 90.80 | | 100 | 0.07 |
| Magnesium | 42001.00 | 37250.00 | 41100.00 | 46300.00 | 20000.00 | | 76600.00 | | 100 | 4.15 |
| Manganese | 17.25 | 13.30 | 16.20 | 20.10 | 0.30 | | 44.30 | | 100 | 0.42 |
| Mercury | 5.20 | 1.61 | 3.20 | 6.36 | 0.21 | | 132.00 | | 97 | 0.30 |
| Selenium | 254.80 | 219.50 | 248.00 | 279.50 | 147.00 | | 1010.00 | | 100 | 1.73 |
| Zinc | 10510.00 | 9355.00 | 10400.00 | 11600.00 | 5120.00 | | 26000.00 | | 100 | 8.74 |

Abbreviations: LOD, limit of detection.

**Table S2.** Distribution of vitamins in first trimester plasma for participants with all vitamin measurements and included in at least one of the cord blood peptide hormone analyses (*N*=484).

| Vitamin | Mean | 25^th^ percentile | 50^th^ percentile | 75^th^ percentile | Minimum | Maximum |
| --- | --- | --- | --- | --- | --- | --- |
| B12 (pg/mL) | 521.5 | 386.2 | 489.4 | 593.8 | 106.1 | 3472.0 |
| Folate (ng/mL) | 27.03 | 14.16 | 19.52 | 29.21 | 7.08 | 359.80 |

**Table S3.** Summary of cord blood peptide hormones included in the study.

| Cord blood peptide hormone | Origin | Function |
| --- | --- | --- |
| Adiponectin | Adipose tissue and vascular cells | Energy homeostasis |
| Leptin | Adipose tissue, placenta | Energy homeostasis |
| IGF-1 | Liver, placenta (regulated by genetics) | Regulates fetal, placental, and neonatal growth |
| IGF-2 | Liver, placenta (regulated by environmental and genetic factors) | Regulates fetal, placental, and neonatal growth |
| IGFBP-3 | Liver, placenta | Limit bioactivity of IGFs; independent role in fetal growth |
| Insulin | Pancreatic β-cells | Regulates fetal growth; stimulates release of IGF-1/2 in response to glucose |
| C-peptide | Pancreatic β-cells | Reflects insulin-secretory activity of pancreatic β-cells, which modulates fetal growth |

Abbreviations: IGF, insulin-like growth factor; IGFBP-3, insulin-like growth factor binding protein.

**Table S4.** Quantile g-computation estimates for difference in each outcome for a one quartile increase in the essential metal mixture, including plasma folate and vitamin B12.

| Outcome  [β (95% bootstrap CI)] | *N* | Essential metal mixture^a^ |
| --- | --- | --- |
| Adiponectin (μg/ml) | 448 | -1.23 (-2.49, 0.04) |
| C-peptide (ng/ml) | 467 | 0.11 (-0.00, 0.21) |
| Insulin (μU/ml) | 467 | 0.69 (-0.53, 1.90) |
| IGF-1 (ng/ml) | 467 | **5.35 (1.52, 9.19)** |
| IGF-1 (ng/ml)^b^ | 467 | 2.66 (-0.27, 5.58) |
| IGF-2 (ng/ml) | 467 | 13.95 (-1.86, 29.76) |
| IGF-2 (ng/ml)^b^ | 467 | 2.64 (-8.70, 13.97) |
| IGFBP-3 (ng/ml) | 467 | **54.36 (0.38, 108.33)** |
| Leptin (ng/ml) | 443 | 0.78 (-0.35, 1.91) |

Abbreviations: CI, confidence interval; IGF, insulin-like growth factor; IGFBP, insulin-like growth factor binding protein; *N*, sample size.

^a^Quantile g-computation model assessed difference in each outcome for one quartile increase in essential metals (magnesium, manganese, selenium, zinc, folate, vitamin B12), conditional on the covariates maternal age, pre-pregnancy body mass index, race and ethnicity, education, household income, smoking status, parity, child sex, the nonessential metals (arsenic, barium, cadmium, cesium, lead mercury), and whether hemolysis and/or lipemia was observed in plasma sample.

^b^Quantile g-computation model assessed difference in each outcome for one quartile increase in essential metals (magnesium, manganese, selenium, zinc, folate, B_12_), conditional on the covariates maternal age, pre-pregnancy body mass index, race and ethnicity, education, household income, smoking status, parity, child sex, the nonessential metals (arsenic, barium, cadmium, cesium, lead, mercury), whether hemolysis and/or lipemia was observed in plasma sample, and IGFBP-3.

**Table S5.** Multivariate linear regression results for association between first trimester red blood cell concentrations of each metal and IGF-1 and IGF-2, additionally adjusted for IGFBP-3.

| Metal^a^ | IGF-1 (ng/ml)  [β (95% CI)]^b^ | IGF-2 (ng/ml)  [β (95% CI)]^b^ |
| --- | --- | --- |
|  | *N*=674 | *N*=674 |
| Arsenic | -0.60 (-1.51, 0.31) | -1.22 (-4.81, 2.36) |
| Barium | 0.78 (-0.24, 1.80) | 0.14 (-3.88, 4.16) |
| Cadmium | 0.09 (-1.17, 1.36) | -4.20 (-9.17, 0.77) |
| Cesium | -0.44 (-3.18, 2.31) | -6.45 (-17.23, 4.34) |
| Lead | -0.25 (-2.46, 1.97) | -3.25 (-11.96, 5.45) |
| Magnesium | 0.68 (-4.32, 5.68) | -3.47 (-23.13, 16.18) |
| Manganese | 0.88 (-0.92, 2.67) | 6.36 (-0.68, 13.41) |
| Mercury | -0.26 (-1.15, 0.62) | -2.24 (-5.70, 1.23) |
| Selenium | 1.18 (-3.52, 5.88) | -5.44 (-23.92, 13.03) |
| Zinc | 0.01 (-5.08, 5.11) | -10.16 (-30.17, 9.86) |

Abbreviations: CI, confidence interval; IGF, insulin-like growth factor; IGFBP, insulin-like growth factor binding protein; *N*, sample size.

^a^Metals were log_2_-transformed in the analyses.

^b^Linear regression models were adjusted for maternal age, pre-pregnancy body mass index, race and ethnicity, education, income, smoking during pregnancy, parity, child sex, and IGFBP-3.

**Table S6.** Multivariate linear regression results for association between plasma concentrations of each vitamin and IGF-1 and IGF-2, adjusting for IGFBP-3.

| Vitamin^a^ | IGF-1 (ng/ml)  [β (95% CI)]^b^ | IGF-2 (ng/ml)  [β (95% CI)]^b^ |
| --- | --- | --- |
|  | *N*=467 | *N*=467 |
| B12 | 2.01 (-1.00, 5.01) | -8.20 (-19.57, 3.17) |
| Folate | 0.61 (-1.31, 2.52) | 1.61 (-5.63, 8.85) |

Abbreviations: CI, confidence interval; IGF, insulin-like growth factor; IGFBP, insulin-like growth factor binding protein; *N*, sample size.

^a^Vitamins were log_2_-transformed in the analyses.

^b^Linear regression models were adjusted for maternal age, pre-pregnancy body mass index, race/ethnicity, education, income, smoking during pregnancy, parity, child sex, whether hemolysis and/or lipemia was observed in plasma sample, and IGFBP-3.

2128 live births

[11 lost to follow-up, 195 withdrew, 7 not specified]

2100 maternal-infant IDs included in present analyses

[kept first live birth only, for participants with more than one live birth]

1390 participants with all first trimester metals measured in red blood cells

[no mercury measurement for 17 participants]

940 participants with first trimester vitamin B12 and folate in plasma

484 participants with at least one cord blood hormone measured

695 participants with at least one cord blood hormone measured

**Figure S1.** Exclusion criteria for mother-infant pairs in Project Viva and final sample size for metals, vitamins, and umbilical cord blood hormones.


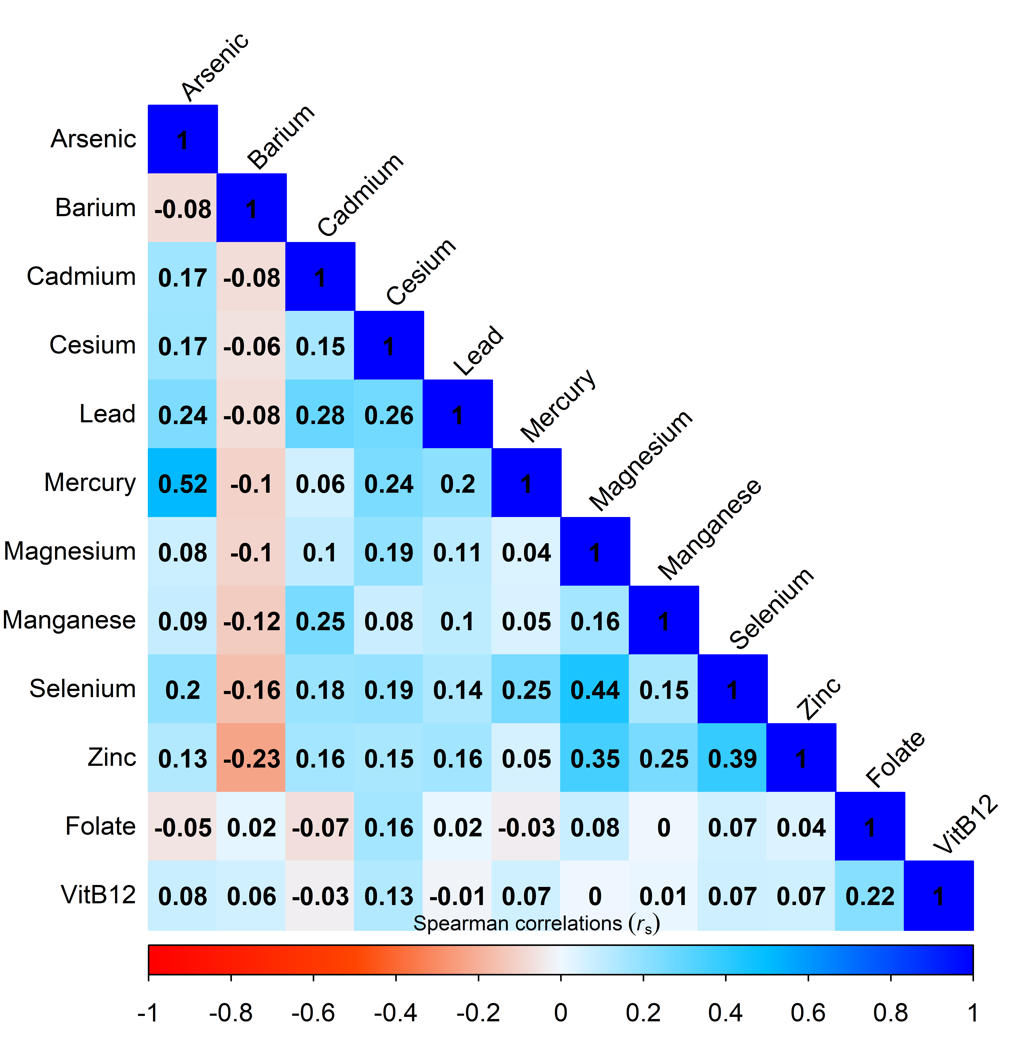


**Figure S2.** Spearman correlation coefficients among prenatal first trimester metals and vitamins (*N*=940)**.**

**
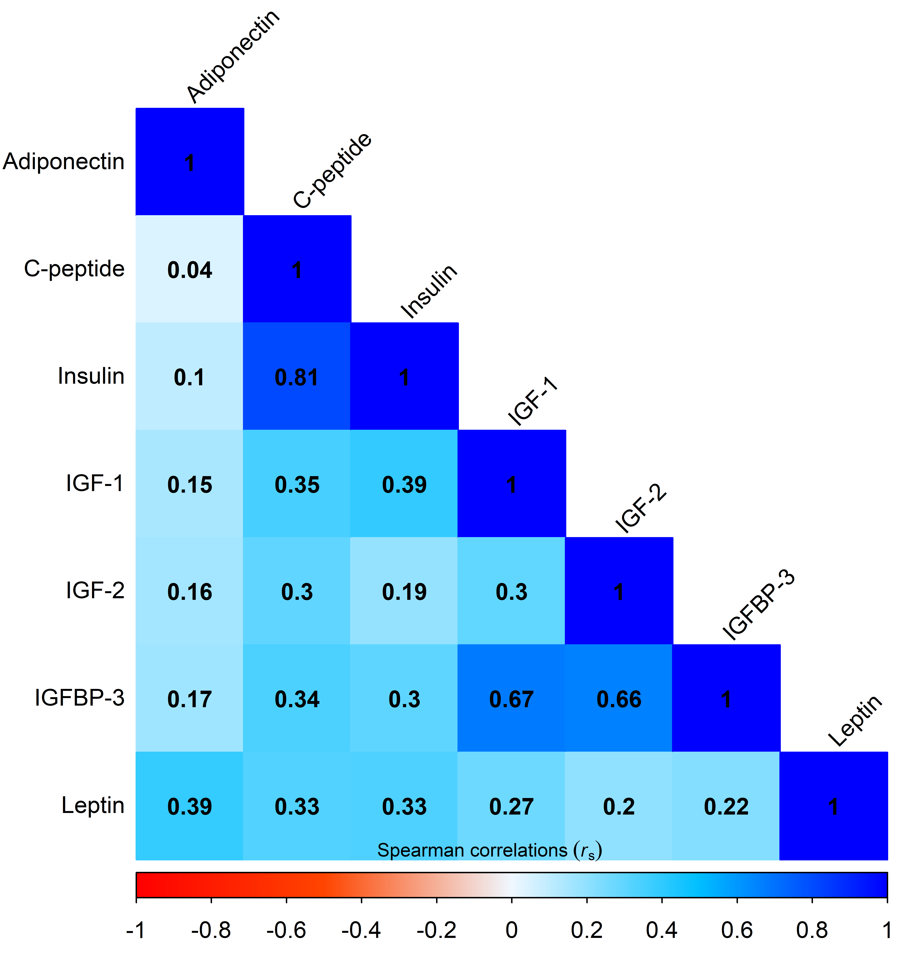
**

**Figure S3.** Spearman correlation coefficients among umbilical cord blood hormones (*N*=570)**.**


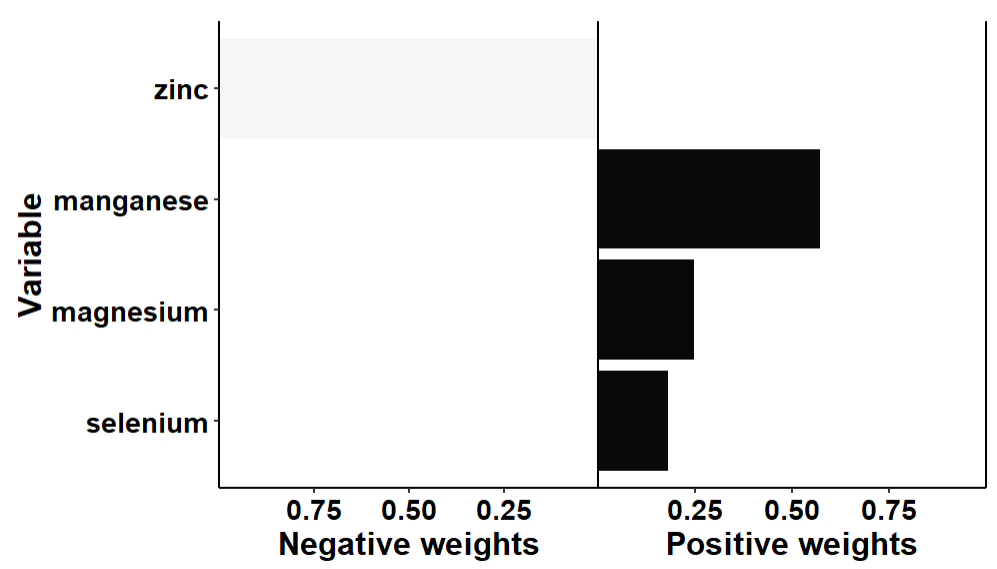


**A**

**B**

**
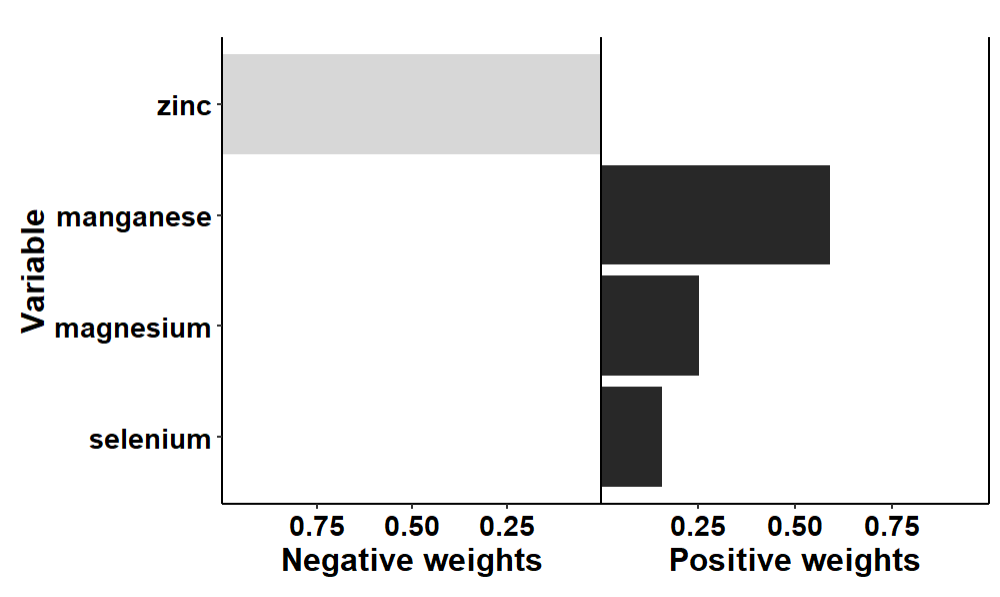
**

**
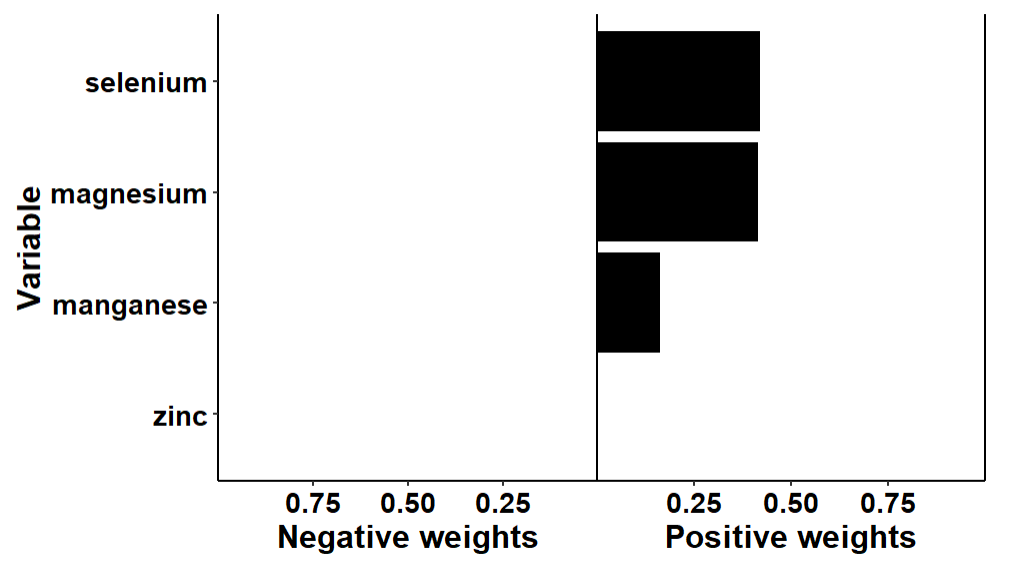
**

**C**

**Figure S4.** Weights representing the proportion of the positive and negative partial effect of each metal in quantile g-computation models examining associations between the essential metal mixture with (A) cord blood IGF-1 (ng/ml), (B) cord blood IGF-2 (ng/ml), and (C) cord blood leptin (ng/ml). Abbreviations: Mg, magnesium; Mn, Manganese; Se, selenium; Zn, zinc. This figure corresponds to models presented in Table 3, without adjustment for IGFBP-3.
